# Contemporary use of excimer laser in percutaneous coronary intervention with indications, procedural characteristics, complications and outcomes in a university teaching hospital

**DOI:** 10.1136/openhrt-2020-001522

**Published:** 2021-04-16

**Authors:** Muhammad Jawad-Ul-Qamar, Harish Sharma, Vincenzo Vetrugno, Kully Sandhu, Peter F Ludman, Sagar N Doshi, Jonathan N Townend, Mohammed Osheiba, Alex Zaphiriou, Sohail Q Khan

**Affiliations:** 1 Department of interventional cardiology, Queen Elizabeth Hospital Birmingham, Birmingham, UK; 2 Institute of Cardiovascular Sciences, University of Birmingham, Birmingham, UK; 3 Cardiology Division, Azienda Ospedaliero-Universitaria di Modena Ospedale Civile di Baggiovara, Modena, Italy

**Keywords:** atherosclerosis, coronary stenosis, percutaneous coronary intervention

## Abstract

**Background:**

Excimer laser coronary atherectomy (ELCA) can be used as an adjunctive percutaneous coronary intervention treatment for challenging, heavily calcified lesions. Although previous studies have documented high rates of complication and restenosis, these predate the introduction of the smaller 0.9 mm laser catheter. As the coronary complexity has increased, there has been a renewed interest in the ELCA. This study investigates the indications, procedural characteristics, complications and outcomes of ELCA in a contemporary coronary interventional practice.

**Methods:**

This single-centre study retrospectively analysed 50 patients treated with ELCA between January 2013 and January 2019.

**Results:**

Patients had a mean age of 67.9±11.4 years with a male predominance (65.3%). 25 (50%) cases were performed in patients with stable angina. Failure to deliver the smallest available balloon/microcatheter was the most frequent indication in 32 (64%) cases for ELCA use. 30 (60%) of the procedures were performed via radial access. The 0.9 mm X-80 catheter was used in 41 (82%) of cases, delivering on average 9000±3929 pulses. ELCA-related complications included 2 coronary dissections and 1 perforation, all of which were covered with stents. No major complications could be directly attributed to the use of ELCA. There was one death and one case of stent thrombosis within 30 days of the procedure.

**Conclusion:**

ELCA can be performed safely via the radial approach with a 0.9 mm catheter with a high success rate by suitably trained operators. The low procedure-related complications with contemporary techniques make this a very useful tool for complex coronary interventions, especially for difficult to dilate lesions and chronic total occlusion vessels.

**Key questions**

**What is already known about this subject?**

- Coronary laser atherectomy is a useful but underused treatment modality in treatment of calcified difficult to cross lesions.
- There have been mixed earlier reports of success with this modality and relatively high procedural complications mostly with the bulky 1.4 mm laser catheter.

**What does this study add?**

- Our work shows that laser atherectomy via radial approach using 0.9 mm laser catheter is safe and convenient with good periprocedural outcomes.

**How might this impact on clinical practice?**

- These results are expected to encourage other centres involved in complex coronary intervention to take up coronary laser atherectomy more frequently in calcified, difficult to dilate lesions and chronic total occlusions.

## Introduction

Percutaneous coronary intervention (PCI) in patients with heavily calcified vessels is challenging. Severely calcified lesions account for up to 12% of patients undergoing coronary angiography^1^ and can prevent the crossing of balloons and even wires. Even if the lesion can be crossed with a wire, calcified or heavily fibrotic plaques may resist high-pressure non-compliant balloon inflations and cutting balloons. Attempting stent implantation within such lesions risk stent malapposition, underdeployment and increase risk of coronary dissection, perforation and stent thrombosis. Specialised techniques and equipment are therefore required for such cases.

Atheroablation by excimer laser coronary atherectomy (ELCA) can be helpful in these circumstances. Excimer lasers use a mixture of rare gas and halogen to generate brief pulses of high-frequency ultraviolet (UV) light. The UV laser can disrupt hard atherosclerotic plaque through several mechanisms, including breaking molecular bonds (photochemical), plaque cell rupture by heat generated at the catheter tip (photothermal) and disruption of intravascular material as vapour bubbles rapidly expand and implode (photomechanical).^2^ The fragments released are small (<10 µm) and thus do not obstruct the coronary microcirculation.^3^ The short wavelength (308 nm) ensures minimal penetration of thermal energy beyond the intended target.

Compared with rotational atherectomy (RA), excimer catheters offer unique advantages including a short monorail segment, easing delivery over a standard 0.014” guidewire. Additionally, the most widely used 0.9 mm X-80 catheter is deliverable via a 6 Fr guide catheter, allowing ELCA to be performed via the radial approach. However, severely calcified plaques may require treatment with both ELCA and RA in combination, referred to as a ‘RASER’ procedure.^4^

Laser atherectomy has been used as an adjunct to percutaneous coronary intervention since the early 1980s.^5^ Early studies have documented high complication rates and restenosis.^6^ However, these studies predate the introduction of the smaller 0.9 mm catheter, which is now the most frequently used laser catheter for coronaries.^7^ Although the efficacy of ELCA has been assessed in a variety of clinical cohorts, there is a paucity of clinical outcome data in a real-world mixture of patients presenting with and without acute myocardial infarction. In this study, we present indications, procedural characteristics, complications and outcomes of ELCA in a contemporary coronary interventional practice in a single large quaternary UK hospital over 6 years.

## Methods

### Study population

Fifty consecutive patients treated with ELCA from 1 January 2013 to 31 January 2019 at the Queen Elizabeth Hospital, Birmingham, UK. Patients were identified using a prospectively maintained British Cardiovascular Interventional Society database.

As it was an all comers cohort, the indications ranged from elective PCI in the setting of stable angina to emergency acute coronary syndromes with cardiogenic shock without any exclusion criteria. Laser was used if the lesion was angiographically assessed as heavily calcified and the inability of the smallest balloon or microcatheter to cross the lesion. For those lesions that were assessed with intravascular ultrasound or optical coherence tomography, laser was used if the imaging suggested a very high thrombus burden or in calcified lesions a ≥270 degree of calcium arc, with ≥0.5 mm thickness and ≥40 mm of length of calcified lesion.

### Outcomes

Immediate procedural success was defined as thrombolysis in myocardial infarction (TIMI) III flow in the target vessel with <50% pre-angioplasty luminal stenosis. Major adverse cardiac and cerebrovascular event (MACCE) were defined as all-cause death, myocardial infarction or stroke and was recorded during hospital stay and at 30-day follow-up.

#### ELCA procedure details

The excimer laser system (Spectranetics CVX-300) uses xenon chloride medium to generate UV light and produces a monochromatic single wavelength—308 nm fluence of 20–80 mJ/mm^2^ and pulse repetition rate of 40–80 Hz. Vascular access was obtained using 6 or 7 Fr sheaths in the radial or femoral arteries. In some cases, both radial and femoral access were required. The laser catheter used was of 0.9 mm or 1.4 mm in diameter. Saline flush and bathe technique was used to clear the blood and dye during delivery of the therapy and the catheter was advanced in small increments with 10 s bursts of lasing.

## Results

### Baseline characteristics

Patients had a mean age of 67.9±11.4 years and a male preponderance 32/50 (64%). The majority of patients (72%) had previous revascularisation with either PCI or coronary artery bypass grafting. Twelve (24%) patients had adjuvant coronary fractional flow reserve/coronary imaging at the same time. Twenty-three (46%) patients were treated electively and 8 (16%) patients were treated as emergencies for ST-elevation MI, among whom 3 (6%) patients were in cardiogenic shock. Laser atherectomy was used as an adjuvant to coronary RA in 11 (22%) cases and thrombus aspiration was performed in 6 (12%) patients. Glycoprotein IIb/IIIa inhibitor was used in eight (16%) cases. Thirty (60%) cases were performed by radial (left/right) approach without the need to change the access site. Only 16 (32%) cases were performed via the femoral approach and a further 4 (8%) used both radial and femoral approach. One of the main indications of ELCA was the failure of balloon tracking due to a calcified lesion or chronic total occlusion (CTO) occurred, which was seen in 32 (64%) cases (table 1).

**Table 1:** Baseline data

Variable	Mean (±SD) number (%)
Age, years	67.9 (±11.4)
Gender	
Male	32 (65.3%)
Female	17 (34.7%)
BMI, kg/m^2^	29.1 (±5.8)
Previous CVA	2 (4.1%)
Peripheral vascular disease	4 (8.2%)
Non-cardiac surgery	2 (4.1%)
Previous MI	23 (46.9%)
Previous CABG	11 (22.4%)
Previous PCI	24 (29%)
Diabetes	
Not diabetic	27 (55.1)
Diet controlled	1 (2)
Oral medicine	12 (24.5)
Insulin dependent	9 (18.4%)
Clinical syndrome	
Stable angina	25 (50%)
UA/NSTEMI	17 (34%)
STEMI	8 (16%)
Cardiogenic shock (consequence of STEMI)	3 (6%)
Target vessel	
LMS/LAD	21 (42%)
LCX/RCA	26 (52%)
SVG	3 (6%)
Procedure urgency	
Elective	23 (46%)
Urgent	19 (38%)
Emergency	8 (16%)
LV function	
Good (EF >50%)	31 (63.3)
Moderate (EF 30%–50%)	8 (16.3)
Poor (EF <30%)	4 (8.2)
Unknown	6 (12.2)
Diagnostic devices	
None	38 (76%)
IVUS/OCT	7 (14%)
Pressure wire	2 (4%)
Procedural indications	
Heavily calcified lesion	22 (44%)
CTO (failure to cross)	10 (20%)
High thrombus burden	11 (22%)
In-stent restenosis	7 (14%)
6 Fr guide catheter	41 (82%)
Arterial access	
Radial (right/left)	30 (60%)
Femoral	16 (32%)
Combined (radial/femoral)	4 (8%)
Glycoprotein IIb/IIIa (eptifibatide)	8 (16%)

BMI, body mass index; CABG, coronary artery bypass grafting; CTO, chronic total occlusion; EF, ejection fraction; IVUS, intravascular ultrasound; LAD, left anterior descending artery; LCX, left cercumflex artery; LMS, left main stem; LV, left ventricle; MI, myocardial infarction; NSTEMI, non-ST-elevation myocardial infarction; OCT, optical coherence tomography; PCI, percutaneous coronary intervention; RCA, right coronary artery; STEMI, ST-elevation myocardial infarction; SVG, saphenous vein grafts; UA, unstable angina.

### Laser technical data

The majority of cases (82%) were performed using the 0.9 mm diameter ELCA catheter. A frequency of 80 Hz was used in 18 (36%) cases and 40–80 Hz in another 12 (24%) cases. The data were not available for the rest of the cases. The mean number of laser pulses were 9000±3929 (table 2).

**Table 2:** Procedural data

Variable	Mean (±SD) number (%)	Variable	Mean (±SD) number (%)
Size of laser catheter (mm)		Fluence	
0.9	41 (82 %)	80	16 (32%)
1.4	3 (6%)	40–80	13 (26%)
Unknown	6 (12%)	Unknown	21 (42%)
Frequency (Hz)		Adjunctive rotational atherectomy	11 (22%)
80	18 (36%)	Size of burr	
40–80	12 (24%)	1.25 mm	4 (8%)
Unknown	20 (40%)	1.5–2 mm	5 (10%)
Number of pulses	9000 (±3929)	>2 mm	2 (4%)

Hz, Hertz; mm, Millimeter.

### Procedure outcomes

**Figure 1:**
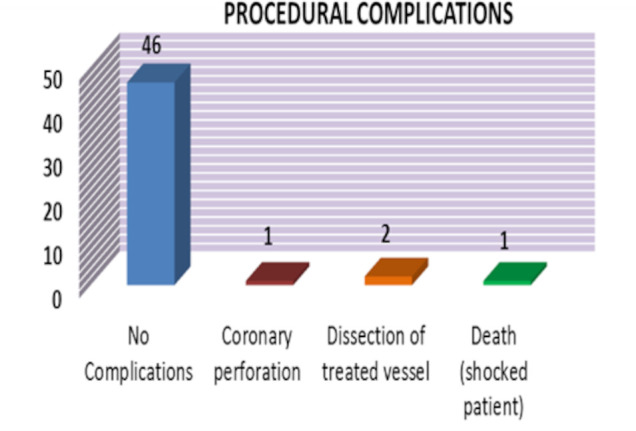
Immediate periprocedural complications while the patient was in the cardiac catheter lab.

There were no periprocedural complications in 46 (92%) patients (figure 1). Laser atherectomy was successful in crossing all uncrossable lesions where the smallest balloon or microcatheter could not cross. A coronary perforation occurred in one case and was treated by a covered stent. The perforation was attributed to use of RA. Two cases of coronary dissection were seen and were treated successfully with a drug-eluting stent. These complications were not directly attributable to the use of ELCA. One death occurred during the procedure in a patient with cardiogenic shock in the context of a late presenting MI. Apart from one mortality, all patients had immediate procedural success with <50% residual stenosis and TIMI III flow in the target vessel. The mean stented segment length was 41 mm (SD 9 mm) and diameter was 3.1 mm (SD 4.2 mm). Within 30 days of the procedure, there was one transient ischaemic attack and one stent thrombosis leading to MI and both these patients made full recovery (table 3).

**Table 3:** 30-Day MACCE (for death, myocardial infarction and stroke/TIA)

Variable	N (%)
No complication	47 (94%)
Death	1 (2%)
Myocardial infarction (subacute stent thrombosis)	1 (2%)
TIA	1 (2%)

MACCE, major adverse cardiac and cerebrovascular event; TIA, transient ischaemic attack.

## Discussion

This study documents the 6-year experience of a single large quaternary centre using ELCA to treat non-crossable, heavily calcified coronary artery lesions. Presence of CTO and very calcified lesion was the main indication in about two-thirds of the cases in our cohort. The results from this retrospective study found that ELCA was associated with high rates of procedural success and low rates of periprocedural complications. The majority of PCI procedures are performed via the radial artery and on finding non-crossable lesions, ELCA can be performed without the need for changing access site. While previous studies report up to 48%^4^ transradial ELCA, in this study 60% of cases were performed via the transradial approach alone without compromising success or complication rate.

This study demonstrates a low rate of coronary dissection compared with other studies. In trials excluding patients with acute MI, the rate of angiographic dissection following ELCA use has been reported as up to 13%.^8^ In another study of 151 post-MI patients treated with ELCA,^9^ major dissection occurred in 5% of cases, compared with only 2% in the present study. Other studies have corroborated that low rates of coronary dissection can be achieved. The multicentre CORAL trial demonstrated that the procedural success of ELCA in the treatment of patients with saphenous vein grafts was lower (82%) than comparative studies in native coronary vessels, however, the rate of major dissection was just under 1% and lower 30-day MACE as compared with control population from theSaphenous vein graft Angioplasty Free of Emboli Randomized (SAFER) trial.^10^

With an ageing population, heavily calcified coronary lesions and in-stent restenosis (ISR) are likely to be encountered with increasing frequency. ELCA is expected to become more widely adopted over time. The use of ELCA to treat patients with ISR has been assessed in several studies. Applying laser to five types of stainless-steel coronary stents did not impact stent endurance or release significant particulate matter. ELCA in the setting of ISR has similar rates of procedural success, complications and long-term clinical outcomes as balloon angioplasty alone,^11^ and RA.^12^ The combination of ELCA and balloon angioplasty produces greater stent expansion than balloon angioplasty alone.^13^

While RA remains the main treatment modality for heavily calcified lesions, it relies on a 0.009-inch RotaWire be advanced distal to the lesion. Case series have demonstrated that RASER can facilitate revascularisation where the RotaWire could not traverse the lesion.^4^ In this study, ELCA was combined with RA in 22% of cases, which is higher than previous work by Badr *et al*.^14^

### Limitations

ELCA is a specialised coronary adjunctive treatment which is performed only in a relatively small number of centres with expertise. This retrospective study is therefore limited by the small number of ELCA procedures performed despite assessing a 6-year timeframe. Furthermore, while 30-day MACCE data are presented, long-term data have not been compiled. Another limitation has been the relatively low use of intracoronary imaging used. This could be explained by the presence of some cases with acute MI and high thrombus burden in which flow restoration and clinical stability was the first priority of management. Moreover, the accepted standard of use of intracoronary imaging in the highly calcified vessels was not as widespread in the earlier half of the dataset.

## Conclusions

ELCA can be performed safely via the radial approach in most patients with a 0.9 mm catheter with a high success rate. The low procedure-related complications with contemporary techniques make this a very useful tool for complex coronary interventions, especially for difficult to dilate lesions or CTO vessels.

## Data Availability

All data relevant to the study are included in the article. Original dataset is available on reasonable request.
